# Functional Loss After Meningitis—Evaluation of Vestibular Function in Patients With Postmeningitic Hearing Loss

**DOI:** 10.3389/fneur.2020.00681

**Published:** 2020-07-30

**Authors:** Niels West, Hjalte Sass, Mads Klokker, Per Cayé-Thomasen

**Affiliations:** ^1^Department of Otorhinolaryngology Head & Neck Surgery and Audiology, Rigshospitalet, University Hospital of Copenhagen, Copenhagen, Denmark; ^2^Faculty of Health and Medical Sciences, University of Copenhagen, Copenhagen, Denmark

**Keywords:** vestibular, hearing loss, cochlear fibrosis, cochlear implant, neuroinfection, vestibulopathy

## Abstract

**Introduction:** The inner ear vestibular system is essential to balance function. Although hearing loss is well-described and quite common following meningitis, the literature evaluating vestibular function following meningitis is very limited. In particular, information on results of contemporary vestibular function tests, e.g., the video head impulse test (VHIT), is scarce. Using contemporary vestibular function tests, this study examines the vestibular function of patients with profound hearing loss (HL) after meningitis.

**Methods:** Review of the literature and retrospective controlled study.

**Patients:** Twenty-one consecutive patients with profound HL after meningitis (cochlear implant candidates) matched with 20 patients with profound HL of unknown etiology and examined during the period 2013–2018.

**Outcome Measure:** Vestibular function loss, as evaluated with VHIT vestibulo-ocular reflex (VOR) gain, eye movement saccades, and cervical vestibular-evoked myogenic potentials (cVEMPs). The results of these tests were correlated to inner ear imaging findings (MRI/CT) and the level of hearing loss.

**Results:** Mean VHIT gain was 0.48 in the meningitis group compared to 0.86 in the control group (*p* < 0.01). Saccades were present in 21 ears (62%) in the meningitis group compared to six ears (15%) among the controls (*p* < 0.01). cVEMP responses were present on five ears (18%) in the meningitis group and 25 ears (66%) in the control group (*p* < 0.01).

**Discussion:** Postmeningitic hearing loss is associated with poor vestibular function, as evaluated by VHIT, saccades, and cVEMP. Loss of vestibular function correlates with the degree of hearing loss and inner ear imaging findings, although not in all cases. Vestibular function should be examined in patients surviving meningitis with hearing loss in order to individualize rehabilitation and improve balance outcome.

## Introduction

Sensorineural hearing loss (SNHL) is the most common complication to bacterial meningitis, affecting more than 50% of survivors of pneumococcal meningitis (1). Unilateral or bilateral profound SNHL has been reported to affect 13% of these individuals, thus being candidates for hearing rehabilitation by a cochlear implant (CI) (1). The vestibular system is located adjacent the cochlea in the inner ear and is essential to balance function. As for hearing loss, loss of vestibular system function can be caused by meningitis, supposedly by the same pathophysiological mechanisms, assumed to be inner ear invasion of bacteria, and subsequent inflammatory infiltration. Rasmussen et al. found that 13 of 90 patients (14%) who survived pneumococcal meningitis demonstrated vestibular areflexia in one or both ears, as determined by bithermal caloric irrigation (2). In addition, vestibular loss was associated with hearing loss, as 9 of 10 patients with postmeningitic deafness suffered from vestibular areflexia on the ipsilateral ear. In a population of bilateral vestibulopathy, meningitis was reported to account for 5% of etiologies (3). Bilateral loss of vestibular function is associated with severe imbalance problems, decreased physical and social activities, as well as poorer quality of life (3, 4). Contrary to hearing function, very few studies have evaluated and quantified loss of vestibular function as a complication to meningitis. Thus, the current literature regarding contemporary evaluation tools [video head impulse test (VHIT) and vestibular-evoked myogenic potentials (VEMPs)] is scarce. Therefore, the aim of this study is to provide a literature overview and to evaluate the vestibular function in patients with profound hearing loss following meningitis (cochlear implant candidates), using VHIT and cervical vestibular-evoked myogenic potential (cVEMP). The findings are correlated with the degree of hearing loss and inner ear findings on MRI/CT and controlled against a matched group of patients with profound hearing loss of unknown etiology.

## Methods

### Study Design

A retrospective review of the medical records and images of all cochlear implant recipients with profound SNHL following meningitis was performed at our tertiary referral center, which is the only CI center in Eastern Denmark, covering 2.6 million inhabitants. In 2013, VHIT and cVEMP was introduced into the workup protocol for CI candidates. Thus, patients receiving a cochlear implant during the period 2013–2018 were included. The study was approved by the Data Protection Agency and the Patient Safety Authority (with record numbers 2012-58-0004 and 3-3013-2344/1, respectively). Patient consent and ethical approval were not required as per local legislation and national guidelines. Twenty-one meningitis cases were identified. Patients were included if the SNHL was a complication to meningitis. To enable comparative analyses, a control group was selected from the center CI-database. A 1:1 ratio between cases and controls was used: 20 consecutive CI recipients with profound hearing loss of unknown etiology (diagnosed “DLA typus incertus”) and implanted during the same period. Thus, a total of 41 patients were evaluated.

### Vestibular Testing

Vestibular function testing included video head impulse tests (VHITs) and cVEMPs. The VHIT (EyeSeeCam, Interacoustics, Middelfart, Denmark or ICS impulse, Otometrics, Taastrup, Denmark) is performed by applying rapid horizontal head thrusts randomly to each side, with gaze fixation (5). The VHIT outcome for the lateral semicircular canals was determined by the vestibulo-ocular reflex (VOR) median gain value (LVOR gain) and compensatory saccades (covert or overt). VOR vestibulopathy (uni- or bilateral) was defined as an ipsilateral gain value below 0.70 or presence of saccades (5). Complete loss of VOR function was defined as a gain value <0.25. Gain values below zero were defined as 0. CVEMPs evaluate the vestibulo-cervical reflex (and thus the function of the saccule in the vestibule of the inner ear) and were performed by applying air-conducted click sounds of 100 dB nHL to the external ear canal and recording myogenic potentials evoked in the ipsilateral sternocleidomastoid muscle (Eclipse, Interacoustics, Middelfart, Denmark) (6). The outcome was evaluated binarily: presence or absence of a potential. VEMP vestibulopathy (uni- or bilateral) was defined as absence of an ipsilateral potential.

### Data Collection

Data were retrieved on age (at meningitis, at vestibular assessment, and at CI), sex, infectious agent, preoperative hearing, VHIT gain values, saccades, and VEMPs. Hearing was reported in terms of pure tone average of 250, 500, 1,000, and 2,000 Hz (PTA4). In addition, computed tomography scan (CT) and/or magnetic resonance images (MRI) of temporal bones were reviewed for abnormal findings in the inner ear, specifically the vestibular system.

### Literature Review

In addition to the retrospective study, a systematic review of the current literature was performed. The PubMed database was used with the search string “Meningitis AND (vestibular dysfunction OR vestibulopathy OR vestibular deficit OR vestibular hypofunction OR vestibular areflexia).” The search was limited to English literature, original studies, and human subjects. Literature from 1990 until today was considered. The retrieved literature is displayed in Table 1.

**Table 1:** Literature on vestibular function after meningitis between 1990 and today, with information on number of meningitis cases that underwent vestibular assessment, and the outcomes of the vestibular investigations.

**References**	***N***	**Vestibular tests employed**	**Results** **(post-meningitis cases)**
Present study	21	VHIT, VEMP	VHIT: mean gain, 0.49/0.47; BVL, 53%; UVL, 29%; saccades, 62%; VEMP: 93% VL (BA = 10; UA = 3)
Levo et al. (7)	7	MHIT	mHIT mean gain 0.28/0.25
Thierry et al. (8)	2	Caloric, HIT, VEMP	Partial VL = 1; Severe/complete VL = 1
Cushing et al. (9)	11^∧^	Caloric, rotation, VEMP	Caloric: BA = 9; UW = 1; rotation VL, 8/10; VEMP: 45% VL (BA = 3; UA = 2)
Wiener-Vacher et al. (10)	34	Caloric, rotation, HIT, VEMP, walking	Complete VL = 44%; partial VL = 32%; associated with SNHL
Cushing et al. (11)	9	Caloric, rotation, VEMP, dynamic balance	Partial or complete VL = all (different between tests; BA = 5; rotation, VL = 7 of 8; VEMP, VL = 3)
Zingler et al. (12)	13^*^	Caloric	All BV
Navacharoen et al. (13)	12^x^	Caloric	BA = 4; UA = 2; associated with SNHL
Rinne et al. (14)	6	Caloric	All BV; all SNHL
Hugosson et al. (15)	22	Caloric	VL = 7 (BA = 2; UA = 1), associated with SNHL
Rasmussen et al. (2)	90	Caloric	14% with BA or UA; associated with SNHL

∧ *Same institution as (11)*.

* *CNS infections*.

x *Fourteen of 19 patients with streptococcus suis meningitis were tested*.

*BA, bilateral areflexia/absent; BVL, bilateral vestibular loss; HIT, head impulse test; mHIT, motorized HIT; SNHL, sensorineural hearing loss; UA, unilateral areflexia/absent; UVL, unilateral vestibular loss; UW, unilateral weakness; VEMP, vestibular-evoked myogenic potential; VL, vestibular loss*.

### Statistics

Data were analyzed using IBM SPSS Statistics software version 22.0 (Armonk, New York, USA) for Windows. Mann–Whitney tests were used to compare groups. Chi-square (χ^2^) or Fisher's exact tests were calculated for correlation analysis of categorical data and Spearman coefficients (*r*_s_) were calculated for numeric data, defining relationship strength as the closer to 1, the stronger the correlation. Significance level was set to *p* < 0.05, and *p*-values were two-tailed.

## Results

Three patients in the meningitis group were excluded since their vestibular assessment data were unavailable, leaving 18 meningitis subjects, 8 female and 10 male. Mean age at meningitis was 21 years (range, 1–61), and mean age at implantation (coinciding with vestibular assessment) was 43 years (range, 14–65). The mean duration of hearing loss (time from meningitis to vestibular assessment) was 26 years (0–63). The infectious agent were *Streptococcus pneumoniae* (eight cases, 44%), *Neisseria meningitidis* (two cases, 11%), viral (one case, 6%) and unknown or unavailable (seven cases, 39%). In the non-meningitis group, 10 were female and 10 were male. Mean age at implantation/vestibular assessment was 69 years (range, 43–84). The group was characterized by non-congenital progressive hearing loss; thus, individual duration of profound hearing loss was not specified for each patient. Patient demographics and audiovestibular results are summarized in Table 2.

**Table 2:** Clinical and audiovestibular characteristics of patients with profound postmeningitic hearing loss and patients with profound hearing loss of unknown origin (DLA typus incertus; control group).

**Age Vest.exam**	**Agent**	**PTA_**4**_** **(dB) R**	**PTA_**4**_** **(dB) L**	**VEMP** **R**	**VEMP** **L**	**VOR** **Gain R**	**VOR** **Gain L**	**Saccades** **R/L**
**PATIENTS WITH PROFOUND POSTMENINGITIC HEARING LOSS**								
37	PC	90	120	0	0	0	0	0/0
57^¤^	NS	120	115	^*^	^*^	0	0	Bilateral overt
56^¤^	NS	120	120	^*^	^*^	0.13	0.08	Bilateral overt
61	PC	120	120	0	0	0.7	0.25	0/overt
57	PC	120	65	0	0	NR	NR	NR
24	MC	85	120	0	0	0.16	0.06	Bilateral covert
40	NS	120	110	0	0	0.02	0.63	Bilateral covert and overt
59^¤^	PC	120	120	0	0	0.19	0.5	Bilateral overt
64	NS	120	120	^*^	^*^	0.29	0.04	Bilateral covert and overt
34	NS	105	120	0	0	0.21	0.34	Bilateral covert
59	PC	45	50	NR	NR	0.26	0	Bilateral covert
14^¤^	PC	100	85	0	1	1.1	1.4	0/0
58	NS	115	90	0	0	0.47	1.03	Covert and overt/0
21	MC	85	120	1	0	0.79	0.58	Bilateral covert and overt
24^¤^	NS	110	90	0	1	0.99	1.05	0/0
61	V	120	95	0	0	1.24	0.99	0/0
66	PC	49	56	1	1	0.73	0.5	0/0
34	PC	120	120	0	0	1.05	0.61	0/covert
**PATIENTS WITH PROFOUND HEARING LOSS OF UNKNOWN ORIGIN**								
68^¤^	–	120	115	0	1	1	1.01	0/0
69	–	70	109	1	1	0.3	0.48	overt/0
73	–	118	104	1	1	0.61	0.76	Bilateral overt
75	–	85	95	1	1	0.62	0.77	0/0
78	–	91	99	1	1	0.65	0.81	overt/0
43	–	108	106	1	1	0.65	0.67	0/0
76	–	73	118	0	0	0.7	0.77	Bilateral covert
84	–	65	74	0	1	0.7	0.61	0/0
79^¤^	–	96	120	1	1	0.74	0.85	0/0
66	–	105	83	1	1	0.75	0.75	0/0
60	–	94	108	1	1	0.77	0.78	0/0
71	–	103	98	0	0	0.79	0.95	0/0
72	–	98	99	1	0	0.82	0.84	0/0
58	–	105	108	1	1	1.01	0.99	0/0
84	–	81	71	0	0	1.03	1.12	0/0
68	–	80	79	0	1	1.04	1.01	0/0
58	–	68	115	1	1	1.08	1.04	0/0
64	–	94	93	0	0	1.1	0.94	0/0
62^¤^	–	65	76	0	0	1.15	0.99	0/0
71	–	114	101	1	1	1.42	1.13	0/0

In a few cases, VEMP (marked with ^*^) was not available.

¤ *Vestibular examination was performed between two sequential cochlear implantations*.

*Gain, VHIT lateral VOR median gain at 60 ms; L, left; MC, meningococcal; NS, not specified (data not available); PC, pneumococcal; PTA4, pure tone average 0.5, 1, 2, 4 kHz; R, right; V, viral; Vest.exam, vestibular examination*.

### Video Head Impulse Test

**Figure 1:**
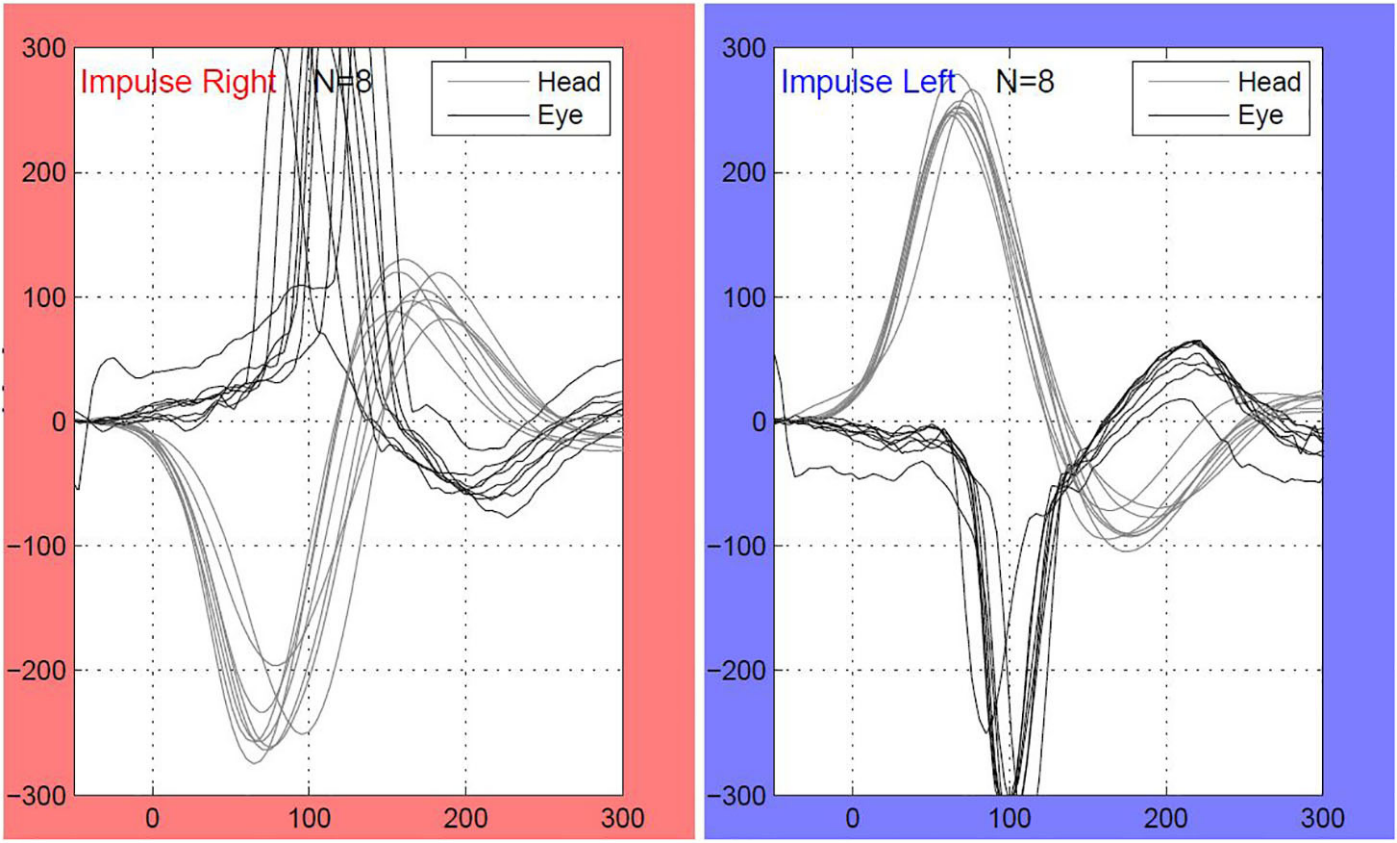
An individual with bilateral deafness after meningococcal meningitis in the childhood. As demonstrated on the video head impulse test (VHIT) of the lateral semicircular canals, the patient has complete loss of vestibulo-ocular reflex (VOR) function. Multiple covert saccades are seen bilaterally. The patient received a cochlear implant (CI) on the right ear. Both CT and MRI were bilaterally normal.

**Figure 2:**
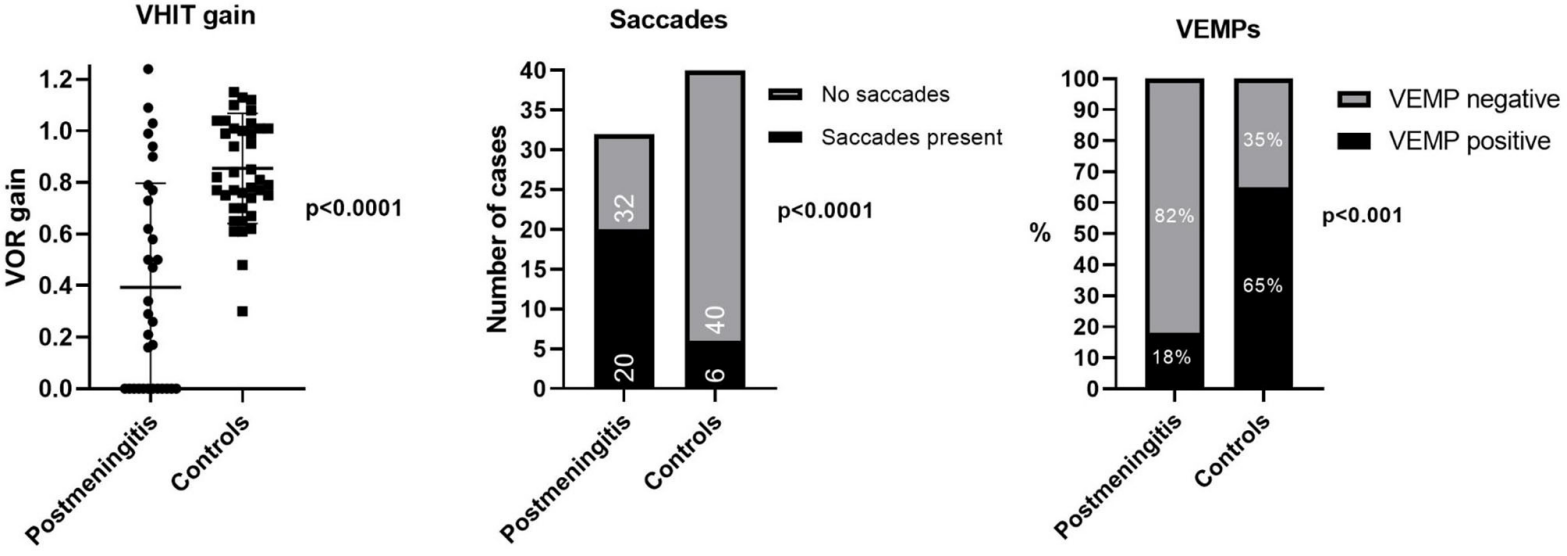
Distribution of lateral VOR gain values, saccades, and cVEMP responses on individual ears according to etiology of hearing loss. In the postmeningitic group, 41% of ears demonstrated a complete loss of VOR function (gain <0.25), and 26% of ears had a normal function (gain >0.75). In the control group, no ears demonstrated complete loss of VOR function, and 68% of ears demonstrated normal function. In addition, the postmeningitic group was dominated by saccades and missing VEMPs. Bars represent mean values with standard deviation. cVEMP, cervical vestibular-evoked myogenic potentials; VOR, vestibulo-ocular reflex.

**Figure 3:**
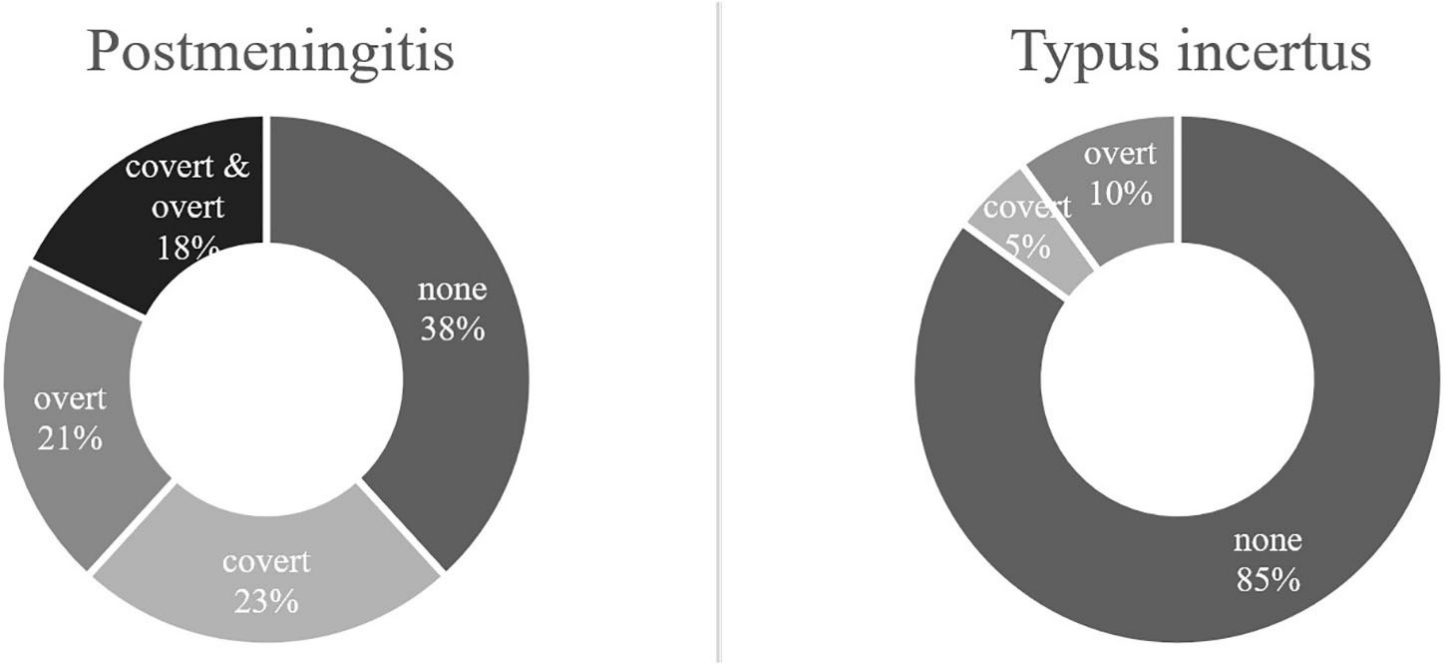
Video head impulse test (VHIT) saccades according to etiology. In the postmeningitic group (*n* = 17), 62% of all ears demonstrated saccades, either as covert (23%), overt (21%), or both (18%). In the control group (*n* = 20), 15% of all ears produced saccades (5% covert, 10% overt). Typus incertus, patients with profound hearing loss of unknown origin.

VHIT outcomes were not available for one patient in the meningitis group, leaving 17 meningitis patients with VHIT results. Mean lateral VOR gain was 0.49/0.47 (right/left) in the meningitis group and 0.85/0.86 in the control group (*p* < 0.0001, Mann–Whitney). Bilateral vestibulopathy occurred in nine cases in the meningitis group (53%) and in two cases (10%) in the control group (*p* < 0.01, χ^2^) (Figure 1). Five patients demonstrated unilateral vestibulopathy in the meningitis group and four patients in the control group. Normal bilateral VOR function was found in three cases in the meningitis group (18%) and in 14 cases in the control group (70%). A complete loss of function on one or two sides was seen in 10 patients (63%) in the meningitis group and was not found among controls. The distribution of gain values according to etiological group is displayed in Figure 2. In the meningitis group, 12 patients (21 ears, 62%) demonstrated saccades (three bilateral covert and overt, three bilateral covert, three bilateral overt, one left-sided overt, one left-sided covert, one right-sided covert, and overt). Four patients in the control group (six ears, 15%) demonstrated saccades (one bilateral covert, one bilateral overt, two unilateral overt) (*p* < 0.0001, χ^2^). In general, saccades were associated with a corresponding abnormal/reduced gain. The distribution of saccades is displayed in Figure 3.

### Cervical Vestibular-Evoked Myogenic Potentials

In the meningitis group, all but four cases had no cVEMPs. Three of these cases were asymmetrical (missing response on one side), and one had bilaterally intact responses. Four patients had no cVEMP testing performed due to missing stapedial reflexes, poor patient cooperation, and an unknown cause. In the control group, 11 patients (55%) demonstrated bilateral cVEMPs, 5 patients (25%) demonstrated no responses bilaterally, and 4 patients (20%) had a unilateral response. Considering the ears individually, 5 ears (18%) had positive cVEMPs and 23 ears (82%) had absent cVEMPs in the meningitis group, compared to 26 positive cVEMPs (65%) and 14 absent cVEMPs (35%) in the control group (*p* < 0.001, χ^2^) (Figure 2).

### Correlation Between Hearing Loss and Vestibular Loss

The last available (preoperative) hearing test was correlated with VHIT gain values on both sides. Pure-tone averages (PTA4) were correlated with the corresponding gain values, and correlation analysis revealed no association for both groups (*r*_s_ = −0.23 and 0.02, *p* = 0.19 and *p* = 0.89). Considering PTA and cVEMP results, the meningitis ears with positive cVEMPs had a mean PTA4 of 73 dbHL, whereas meningitis ears with absent cVEMPs had a mean PTA4 of 110 dbHL (*p* = 0.002, Mann–Whitney). In the control group, ears with positive cVEMPs had a mean PTA4 of 99 dbHL, and ears with absent cVEMPs had a mean PTA4 of 89 dbHL (*p* = 0.07, Mann–Whitney).

### Inner Ear Imaging

**Figure 4:**
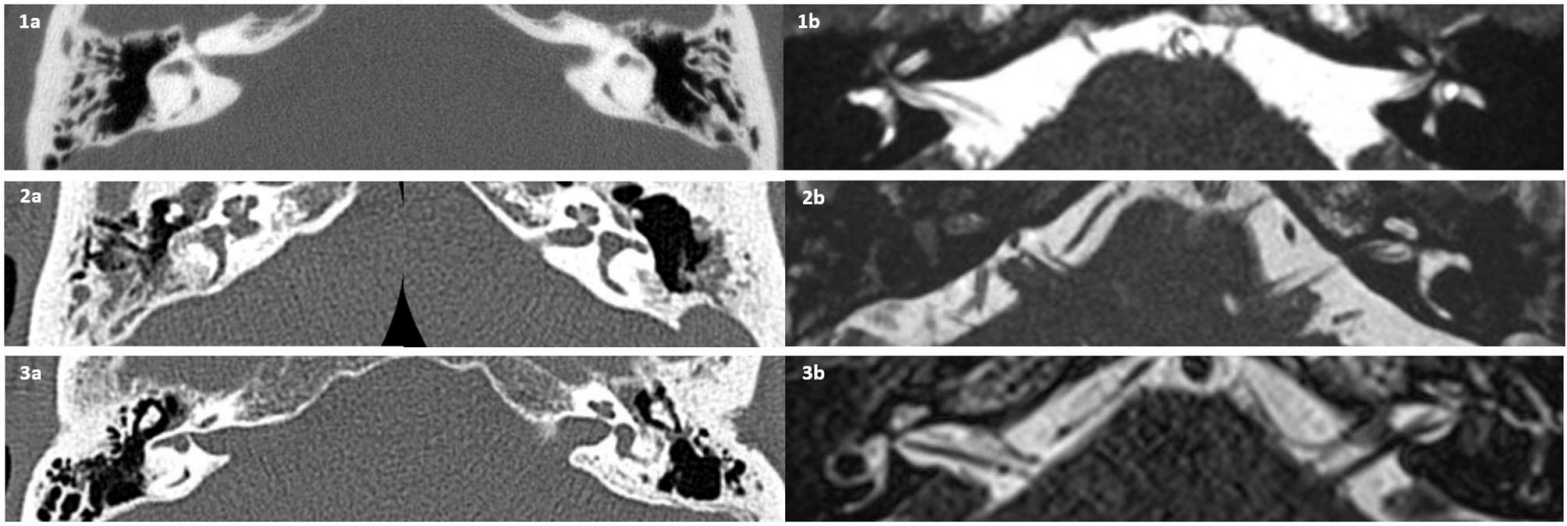
Preoperative temporal bone computed tomography (CT) and magnetic resonance imaging (MRI), axial plane. **(1)** Pneumococcal meningitis. **(1a)** CT showing bilateral sclerotic foci in lateral semicircular canal (L-SCC). **(1b)** MRI showing bilateral hypointense L-SCC fluid signals. The patient demonstrated absent vestibular-evoked myogenic potentials (VEMPs) and complete loss of vestibulo-ocular reflex (VOR) function. **(2)** Pneumococcal meningitis. **(2a)** Right-sided L-SCC ossification on CT; left L-SCC normal. **(2b)** Absent MRI fluid signal of right labyrinth; left side appears normal. The patient had bilateral sensorineural hearing loss, anacusis on right side and PTA 65 left, and bilateral missing cVEMPs. **(3)** Pneumococcal meningitis and following bilateral cochlear implant (CI). **(3a)** CT showing normal appearance of the right L-SCC, in contrary to the left L-SCC, which appears irregular, interpreted as ossification. **(3b)** Decreased left-sided MRI fluid signal and normal on right side. The patient had bilaterally missing cVEMPs, however unilateral VHIT deficit (complete loss of VOR on left side).

**Figure 5:**
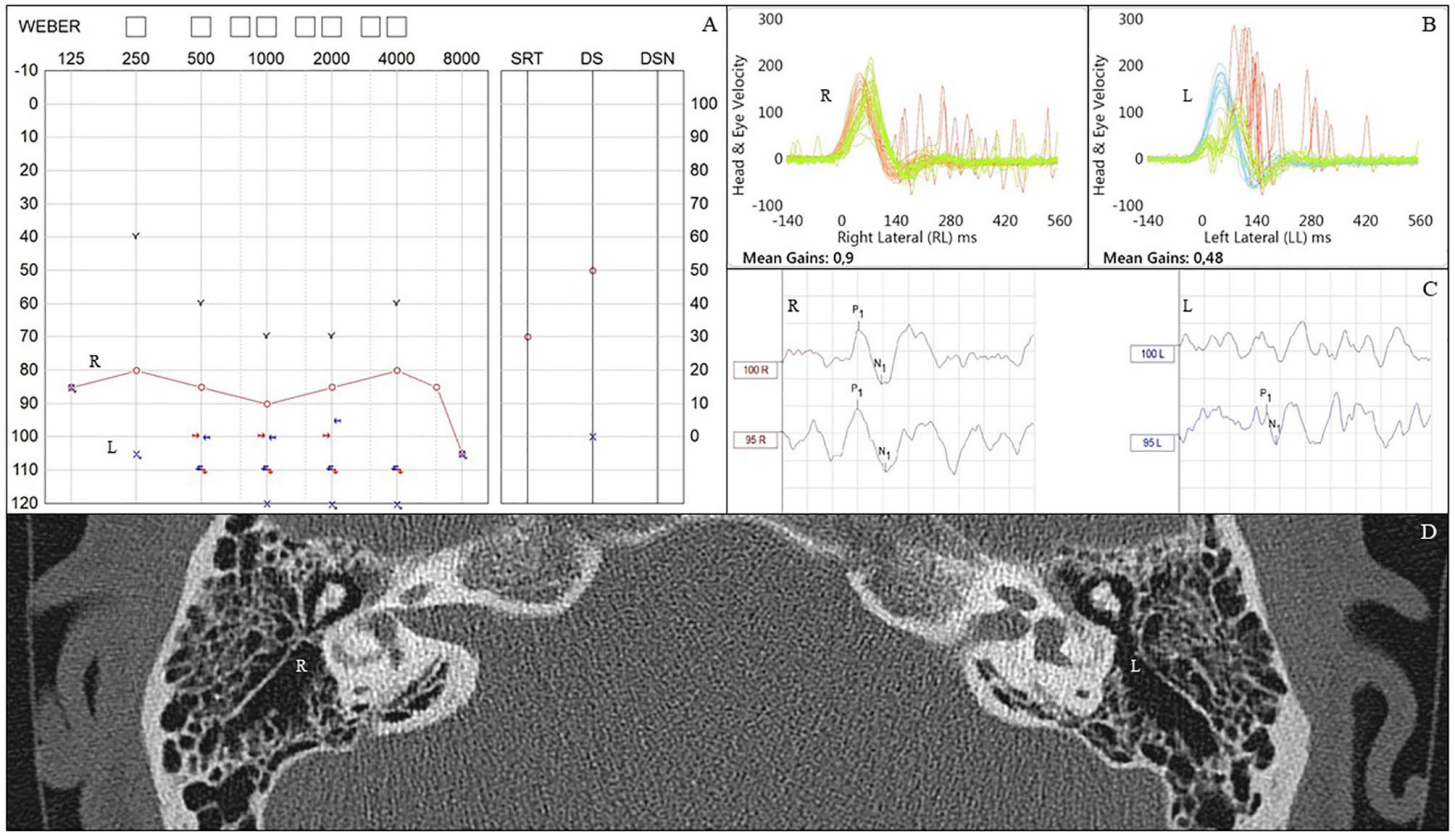
Audiovestibular findings in an individual who had bacterial meningitis in the childhood. **(A)** Pure tone audiometry demonstrating right-sided profound hearing loss and left-sided anacusis. **(B)** Video head impulse test (VHIT) results showing normal gain (0.9) on the right side and abnormally low gain (0.48) on the left side, where covert and overt refixation saccades are also seen. **(C)** Cervical vestibular evoked myogenic potentials (cVEMPs) are elicited on right side but not on the left. **(D)** Computed tomography scan demonstrating signs of ossification in the left lateral semicircular canal. Right side appears normal. L, left; R, right; VHIT, video head impulse test.

In the meningitis group, all patients had either an available temporal bone CT or MRI. Six patients (33%) had bilaterally normal configuration of their vestibular apparatus, whereas four patients (22%) demonstrated a partial or complete bilateral vestibular system fluid signal decrease (MRI T2W), and eight patients (44%) demonstrated a unilateral signal decrease (Figure 4). Comparing fluid signal change (side specific) and vestibular loss on VHIT (gain <0.70 and/or saccades), there was a clear association between vestibular deficit and imaging pathology (*p* < 0.01, χ^2^). Two cases of bilateral vestibulopathy had normal radiological findings, and another six ears demonstrated discrepancy between vestibular function and radiological appearance. This relation was not found for cVEMP responses (*p* = 0.55, Fisher's exact). In the control group, 16 patients had a posterior fossa MRI, and three patients had a CT. All cases in the control group had bilateral normal appearance of the vestibular apparatus. Figure 5 illustrates a 21-year-old patient with asymmetrical audiovestibular test results supported by asymmetrical radiological findings.

## Discussion

### Occurrence of Vestibular Dysfunction in Patients With Profound Post-meningitic Hearing Loss

This study is the largest series evaluating vestibular function in patients with profound hearing loss after meningitis and the first to include both VHIT and VEMPs. The study demonstrates that the VHIT can be applied to detect and quantify meningitis-induced vestibular loss. Our findings also show that unilateral vestibulopathy (UVP) and bilateral vestibulopathy (BVP) are commonly occurring among patients with profound SNHL as sequelae to meningitis. This is in great contrast to our control group of non-meningitis patients with profound hearing loss, who despite older age performs significantly better according to all vestibular parameters.

Eight patients were evaluated between two sequential CI operations, representing a limitation of the study since ipsilateral loss of vestibular function is a known risk of implantation, particularly when evaluated by the head impulse test (16, 17). However, implantation did not appear to have any effect on vestibular findings in these cases, and there was a similar number of sequential CI receivers in each group (five in the meningitis group and three in the non-meningitis group). Thus, the degree of vestibular loss among the sequential CI receivers was comparable to that of the first-time CI receivers.

This series of patients did not include meningitis survivors with normal hearing or those with only mild or moderate hearing loss. It is plausible that vestibular dysfunction is underreported since dysfunction may be present among patients with normal hearing (2). Moreover, as data on all meningitis cases, e.g., those deceased and those recovered including hearing status, were unavailable, epidemiological conclusions cannot be drawn. Consequently, the results in the present study are not generalizable to the all individuals who have recovered from meningitis. Lateral semicircular canal ossification has been reported to occur before cochlear ossification, indicating that affection of the vestibular system may precede that of the cochlea (18–20). In addition, our study is also biased by the fact that indeed purulent meningitis can cause ossification obliterating the cochlea, thus preventing successful electrode insertion (21). If severe cochlear ossification occurs in our setting, workup for implantation is halted. Thus, this patient subgroup was not evaluated in the present study (data not available). However, there is no reason to believe that patients with ossified labyrinths would outperform patients included in present series. Future studies investigating vestibular function in all meningitis survivors are warranted.

In 1998, Rinne et al. (14) published a review of the records of 53 patients with bilateral vestibular failure defined by the absence of nystagmus response to bithermal caloric irrigation. Six cases (11%) were attributed to meningitis (*streptococcus suis*, meningococcus, *S. pneumoniae*, and unknown agent), and all of these had concurrent SNHL. Similarly, in 2009, Zingler et al. (12) retrospectively evaluated 255 patients with bilateral vestibulopathy. Thirteen cases (5%) were caused by meningoencephalitis (meningitis, encephalitis, or cerebellitis). No assessments of hearing were reported.

Hugosson et al. (15) studied a population of 22 adults who had recovered from childhood meningitis. At long-term, bithermal caloric irrigation was performed. Six of nine individuals (67%) with abnormal hearing had an abnormal caloric result, of which two had bilateral and one unilateral areflexia. In the group with normal hearing, one patient had unilateral areflexia, Thus, hearing loss and vestibulopathy were clearly associated. In 12 survivors of streptococcus suis meningitis, Navacharoen et al. (13) reported that 6 (50%) demonstrated vestibular impairment as evaluated by bithermal caloric irrigation. Four cases (33%) demonstrated bilaterally absent caloric reflex and two cases (17%) had unilaterally absent response. Reportedly, the vestibular deficit was associated with profound hearing loss in most cases.

Wiener-vacher et al. (10) performed extensive vestibular testing (bithermal caloric test, rotation test, head impulse test, and VEMP) of 34 children referred with postural instability after meningitis. Twenty-six children (76%) had vestibular impairment (15 complete, 44%; 11 partial, 32%). Vestibular loss had a strong correlation with hearing loss. The study did not account for the consistency in the test battery for each patient. Summarized, vestibular loss was found to affect 11% of patients after bacterial meningitis, of which the half had complete loss of vestibular function (10).

Thierry et al. (8) investigated 43 children after cochlear implantation. Etiology of hearing loss was meningitis in two cases. Vestibular tests consisted of the VEMP, the caloric test, and the head impulse test. The two meningitis cases were tested postimplantation and were reported to have partial vestibular dysfunction and severe dysfunction or areflexia, respectively.

In 2013, Cushing et al. (9) investigated a large pediatric cohort of which 11 children were meningitis survivors. Patients were examined by the caloric test, the VEMP test, and the rotation test. The majority displayed abnormal vestibular function, as 9 of 10 had bilateral caloric areflexia and eight had abnormal response to rotational testing. The VEMP responses (saccular end organ) was the least affected outcome, since only three cases had bilaterally absent response, and two had unilateral responses. The same group published a paper in 2009, assessing nine pediatric patients after bacterial meningitis (11). Interestingly, this study concluded that, due to compensatory mechanisms, vestibular deficits did not necessarily match the subjective perception of handicap. In present study, no clinical data on subjective disequilibrium were available. Future protocols should apply quantifiable subjective measurements [e.g., the Dizziness Handicap Inventory (22)] to specify vertigo complaints further and to correlate objective vestibular findings to subjective outcomes.

All but one (9) of the above studies are retrospective. Inherent limitations of retrospective studies include overlooked cases, underreporting, and omittance of important information. Future studies should apply prospective designs to overcome selection and information bias associated with the retrospective aspect.

### Association Between Vestibular Dysfunction and Hearing Loss After Meningitis

From the studies referenced above, it appears that some association does exist between hearing loss and vestibular loss after meningitis, which makes sense as the inflammation/infection may spread to all compartments of the inner ear (23, 24). However, Levo et al. (7) investigated 23 patients with bilateral vestibular hypofunction as measured with a motorized head impulse test. In seven cases, the etiology was meningitis. Two of these patients had normal hearing. Accordingly, and as also noted above, vestibular dysfunction may occur without hearing loss, whereas the opposite appears to be rare. Thus, the vestibular system may be the first part of the inner ear to be affected in humans, whereas cochlear involvement appears to be first in line in other species (23, 24). This perspective increases the relevance of performing a screening of not only hearing but also vestibular function in all patients surviving meningitis.

### Association Between Vestibular Dysfunction and Findings of Inner Ear Imaging

A previous study found that the occurrence of labyrinthitis ossificans was associated with pneumococcal infection (21). In the present study, the etiological subgroups were too small for statistical evaluation. However, it is remarkable that the present case of viral meningitis demonstrated the best VHIT results (gain, 1.24/0.99). Another study investigated three cases of isolated vestibular ossification after bacterial meningitis that all received CI due to profound SNHL (19). However, vestibular function was not accounted for.

In the present study, we found a clear association between radiological findings and vestibular function. However, some patients had reduced VHIT gain despite normally appearing semicircular canals on CT. On the other hand, one patient displayed fluid signal decrease in the right vestibular apparatus on MRI and ipsilateral normal vestibular function. The patient had no signs of vestibular ossification on CT.

### Vestibular Test Battery

In other vestibular pathologies (e.g., vestibular schwannomas and Ménière's disease), a discrepancy in findings can often be found between VHIT and the caloric test in detecting vestibular loss (25–27). Although investigating the function of the same end organ, the caloric test stimulates low-frequency fibers while the head impulse test stimulates high-frequency fibers (28). With this perspective in mind, adding the caloric test to the test battery may reveal even more patients with vestibular deficits after meningitis. Further studies are warranted to test this hypothesis.

In the present study, the VHIT tested the VOR through stimulation of the lateral semicircular canals and thus the superior vestibular nerve. The cVEMP procedure tests the vestibulo-cervical reflex through stimulation of the saccule and thus the inferior vestibular nerve (6). Elicitation of cVEMPs is known to be influenced by hearing function, as a conductive hearing loss can hamper the elicitation of an ipsilateral VEMP. This potential limitation in testing the inferior vestibular pathway could be overcome by testing of all semicircular canals by the VHIT.

### Vestibular Screening of Meningitis Patients

As found in the present literature review, publications on the field are scarce. It could be argued that more attention should be given to meningitis survivors regarding balance complaints and testing of vestibular function, subsequently vestibular rehabilitation. It is reasonable to suspect that disequilibrium or balance issues among meningitis patients are sometimes neglected and in best case explained by neurological factors (10). Identifying vestibular loss provides information and clarity for the medical professional and the patient alike. In addition, it provides the basis for a vestibular rehabilitation strategy. Thus, it seems reasonable to suggest that all patients recovering from meningitis should undergo screening for loss of not only hearing but also vestibular function. In the future, vestibular implants (29) may also constitute a treatment option for patients with bilateral vestibulopathy after meningitis. Ramos Macias et al. recently demonstrated that otolith organ implantation was feasible in two patients with bilateral vestibulopathy due to meningitis (30). Plausibly, this procedure may have a window of opportunity as for cochlear implantation, due to progressing ossification of the inner ear fluid compartments (21). Thus, rapid referral to screening of both hearing and vestibular function may be important in the future.

## Conclusion

This study investigates vestibular function in terms of VHIT gain, saccades, and cVEMPs in a consecutive series of patients with hearing loss after meningitis. When results are compared with a matched group of patients with profound sensorineural hearing loss of unknown etiology, it is evident that meningitis patients have a high rate of severe vestibular dysfunction, since more than half has complete or near complete loss of vestibular function. Hearing loss is the most common complication following meningitis. As postmeningitic hearing loss is associated with vestibular loss in most cases, we advocate that patients surviving meningitis complicated with hearing loss should undergo not only audiometry but also vestibular testing, in order to improve diagnostic accuracy in case of balance problems and subsequently to design an individual vestibular rehabilitation program.

## Data Availability Statement

All datasets generated for this study are included in the article/supplementary material.

## Ethics Statement

Ethical approval was not required as per local legislation and national guidelines.

## Author Contributions

NW: ideation, data collection, and manuscript preparation. HS: manuscript review. MK: data collection and manuscript review. PC-T: ideation, manuscript preparation, and review. All authors: contributed to the article and approved the submitted version.

## Disclosure

The contents of this paper have not been published previously. Part of the data has been presented at the Confederation of European Otorhinolaryngology—Head & Neck Surgery (CEORL) Conference, Brussels, July 2019.

## Conflict of Interest

The authors declare that the research was conducted in the absence of any commercial or financial relationships that could be construed as a potential conflict of interest.

## References

[1] Worsøe L Cayé-Thomasen P Brandt CT Thomsen J Østergaard C. Factors associated with the occurrence of hearing loss after pneumococcal meningitis. Clin Infect Dis. (2010) 51:917–24. 10.1086/656409 20815735

[2] Rasmussen NJ. Otologic sequelae after pneumococcal meningitis: a survey of 164 consecutive cases with a follow-up of 94 survivors. Laryngoscope. (1991) 101:876–82. 10.1288/00005537-199108000-00012 1865737

[3] Strupp M Feil K Dieterich M Brandt T Bilateral vestibulopathy, 1st ed Vol. 137 Handbook of Clinical Neurology. Elsevier BV (2016). p. 235–40. 10.1016/B978-0-444-63437-5.00017-0 27638075

[4] Guinand N Boselie F Guyot JP Kingma H. Quality of life of patients with bilateral vestibulopathy. Ann Otol Rhinol Laryngol. (2012) 121:471–7. 10.1177/000348941212100708 22844867

[5] Halmagyi GM Chen L MacDougall HG Weber KP McGarvie LA Curthoys IS The video head impulse test. Front Neurol. (2017) 8:258 10.3389/fneur.2017.00258 28649224 PMC5465266

[6] Brantberg K. Cervical vestibular evoked myogenic potentials (cVEMPs): usefulness in clinical neuro-otology. Semin Neurol. (2009) 29:541–7. 10.1055/s-0029-1241042 19834866

[7] Levo H Aalto H Hirvonen TP. Bilateral vestibular hypofunction in quantitative head impulse test: clinical characteristics in 23 patients. J Int Adv Otol. (2017) 13:354–57. 10.5152/iao.2017.4211 29092804

[8] Thierry B Blanchard M Leboulanger N Parodi M Wiener-Vacher SR Garabedian EN. Cochlear implantation and vestibular function in children. Int J Pediatr Otorhinolaryngol. (2015) 79:101–4. 10.1016/j.ijporl.2014.11.002 25500550

[9] Cushing SL Gordon KA Rutka JA James AL Papsin BC. Vestibular end-organ dysfunction in children with sensorineural hearing loss and cochlear implants: an expanded cohort and etiologic assessment. Otol Neurotol. (2013) 34:422–8. 10.1097/MAO.0b013e31827b4ba0 23370550

[10] Wiener-Vacher SR Obeid R Abou-Elew M. Vestibular impairment after bacterial meningitis delays infant posturomotor development. J Pediatr. (2012) 161:246–51.e1. 10.1016/j.jpeds.2012.02.009 22445260

[11] Cushing SL Papsin BC Rutka JA James AL Blaser SL Gordon KA. Vestibular end-organ and balance deficits after meningitis and cochlear implantation in children correlate poorly with functional outcome. Otol Neurotol. (2009) 30:488–95. 10.1097/MAO.0b013e31819bd7c8 19395989

[12] Zingler VC Weintz E Jahn K Huppert D Cnyrim C Brandt T. Causative factors, epidemiology, and follow-up of bilateral vestibulopathy. Ann N Y Acad Sci. (2009) 1164:505–8. 10.1111/j.1749-6632.2009.03765.x 19645958

[13] Navacharoen N Chantharochavong V Hanprasertpong C Kangsanarak J Lekagul S. Hearing and vestibular loss in streptococcus suis infection from swine and traditional raw pork exposure in northern Thailand. J Laryngol Otol. (2009) 123:857–62. 10.1017/S0022215109004939 19275779

[14] Rinne T Bronstein AM Rudge P Gresty MA Luxon LM. Bilateral loss of vestibular function: clinical findings in 53 patients. J Neurol. (1998) 245:314–21. 10.1007/s004150050225 9669481

[15] Hugosson S Carlsson E Borg E Brorson LO Langeroth G Olcén P. Audiovestibular and neuropsychological outcome of adults who had recovered from childhood bacterial meningitis. Int J Pediatr Otorhinolaryngol. (1997) 42:149–67. 10.1016/S0165-5876(97)00129-8 9692625

[16] Ibrahim I da Silva SD Segal B Zeitouni A. Effect of cochlear implant surgery on vestibular function: meta-analysis study. J Otolaryngol Head Neck Surg. (2017) 46:44. 10.1186/s40463-017-0224-0 28595652 PMC5465585

[17] Batuecas-Caletrio A Klumpp M Santacruz-Ruiz S Gonzalez FB Sánchez EG Arriaga M. Vestibular function in cochlear implantation: correlating objectiveness and subjectiveness. Laryngoscope. (2015) 125:2371–5. 10.1002/lary.25299 25891786

[18] Muren C Bredberg G. Postmeningitic labyrinthine ossification primarily affecting the semicircular canals. Eur Radiol. (1997) 7:208–13. 10.1007/s003300050137 9038117

[19] Reeck JB Lalwani AK. Isolated vestibular ossification after meningitis associated with sensorineural hearing loss. Otol Neurotol. (2003) 24:576–81. 10.1097/00129492-200307000-00008 12851548

[20] Chan CC Saunders DE Chong WK Hartley BE Raglan E Rajput K. Advancement in post-meningitic lateral semicircular canal labyrinthitis ossificans. J Laryngol Otol. (2007) 121:105–9. 10.1017/S002221510600377X 17123454

[21] Caye-Thomasen P Dam MS Omland SH Mantoni M. Cochlear ossification in patients with profound hearing loss following bacterial meningitis. Acta Otolaryngol. (2012) 132:720–5. 10.3109/00016489.2012.656323 22497482

[22] Jacobson GP Newman CW. The development of the dizziness handicap inventory. Arch Otolaryngol Neck Surg. (1990) 116:424–7. 10.1001/archotol.1990.01870040046011 2317323

[23] Caye-Thomasen P Worsøe L Brandt CT Miyazaki H Østergaard C Frimodt-Møller N Thomsen J. Routes, dynamics, and correlates of cochlear inflammation in terminal and recovering experimental meningitis. Laryngoscope. (2009) 119:1560–70. 10.1002/lary.20260 19504554

[24] Møller MN Brandt C Østergaard C Caye-Thomasen P. Bacterial invasion of the inner ear in association with pneumococcal meningitis. Otol Neurotol. (2014) 35:e178–86. 10.1097/MAO.0000000000000305 24569797

[25] Jung J Suh MJ Kim SH. Discrepancies between video head impulse and caloric tests in patients with enlarged vestibular aqueduct. Laryngoscope. (2017) 127:921–6. 10.1002/lary.26122 27374754

[26] West N Sass H Klokker M Cayé-Thomasen P. Video head impulse test results in patients with a vestibular schwannoma-sensitivity and correlation with other vestibular system function tests, hearing acuity, and tumor size. Otol Neurotol. (2020) 41:e623–9. 10.1097/MAO.0000000000002600 32118807

[27] Blödow A Blödow J Bloching MB Helbig R Walther LE. Horizontal VOR function shows frequency dynamics in vestibular schwannoma. Eur Arch Oto-Rhino-Laryngol. (2015) 272:2143–8. 10.1007/s00405-014-3042-2 24789061

[28] Perez N Rama-Lopez J. Head-impulse and caloric tests in patients with dizziness. Otol Neurotol. (2003) 24:913–7. 10.1097/00129492-200311000-00016 14600474

[29] Guinand N Van De Berg R Cavuscens S Stokroos RJ Ranieri M Pelizzone M. Vestibular implants: 8 years of experience with electrical stimulation of the vestibular nerve in 11 patients with bilateral vestibular loss. Orl J Otorhinolaryngol Relat Spec. (2015) 77:227–40. 10.1159/000433554 26367113

[30] Ramos Macias A Ramos de Miguel A Rodriguez Montesdeoca I Borkoski Barreiro S Falcón González JC. Chronic electrical stimulation of the otolith organ: preliminary results in humans with bilateral vestibulopathy and sensorineural hearing loss. Audiol Neurotol. (2020) 25(Suppl. 1–2):79–90. 10.1159/000503600 31801137

